# Malignant Pleural Effusion: Diagnosis and Management

**DOI:** 10.1155/2020/2950751

**Published:** 2020-09-23

**Authors:** Lucía Ferreiro, Juan Suárez-Antelo, José Manuel Álvarez-Dobaño, María E. Toubes, Vanessa Riveiro, Luis Valdés

**Affiliations:** ^1^Pulmonology Department, University Clinical Hospital of Santiago, Santiago de Compostela, Spain; ^2^Interdisciplinary Research Group in Pulmonology, Health Research Institute of Santiago de Compostela (IDIS), Santiago de Compostela, Spain

## Abstract

Symptomatic malignant pleural effusion is a common clinical problem. This condition is associated with very high mortality, with life expectancy ranging from 3 to 12 months. Studies are contributing evidence on an increasing number of therapeutic options (therapeutic thoracentesis, thoracoscopic pleurodesis or thoracic drainage, indwelling pleural catheter, surgery, or a combination of these therapies). Despite the availability of therapies, the management of malignant pleural effusion is challenging and is mainly focused on the relief of symptoms. The therapy to be administered needs to be designed on a case-by-case basis considering patient's preferences, life expectancy, tumour type, presence of a trapped lung, resources available, and experience of the treating team. At present, the management of malignant pleural effusion has evolved towards less invasive approaches based on ambulatory care. This approach spares the patient the discomfort caused by more invasive interventions and reduces the economic burden of the disease. A review was performed of the diagnosis and the different approaches to the management of malignant pleural effusion, with special emphasis on their indications, usefulness, cost-effectiveness, and complications. Further research is needed to shed light on the current matters of controversy and help establish a standardized, more effective management of this clinical problem.

## 1. Introduction

Malignant pleural effusion (MPE) is the second most frequent cause of pleural exudate [1]. The presence of malignant cells in the pleural fluid (PF) and/or pleural tissue confirms the presence of disseminated or advanced cancer and is associated with a lower life expectancy. According to a recent study, the survival of lung cancer patients with MPE is 5.5 months [2], whereas overall survival for all types of cancer ranged from 3 to 12 months, based on the type of tumour and comorbidities of the patient [3]. MPE affects 15% of oncologic patients [4]. As many as 50,000 and 150,000 new cases of MPE are diagnosed in UK [5] and USA [6] every year, with hospitalization costs amounting to more than five billion dollars [7]. Most MPE are symptomatic and frequently cause dyspnea, which is not only associated with the size of the PE, but also associated with the rate of fluid accumulation and the presence of underlying cardio-respiratory comorbidities. Other symptoms include chest pain, dry cough, or early feeling of satiety due to the pressure exerted by the PE on the stomach [3].

The management of MPE is merely palliative and should focus on the relief of symptoms. Therefore, recurrent invasive procedures should be avoided [8]. A recent study demonstrated that definitive pleural procedures (pleurodesis, indwelling pleural catheter (IPC), or both) compared to repeat thoracentesis resulted in fewer subsequent pleural procedures, fewer pneumothoraxes, and fewer Emergency Department procedures [8]. In the same line, avoidance of hospitalization and prevention of complications are recommended [9]. Individual patient's preferences should also be considered, together with comorbidities, recurrent PEs, the presence of loculations or a trapped lung, the features of the tumour, or the type of cancer [10].

Recent relevant studies [11, 12] have expanded the traditional portfolio of treatments for MPE [3, 5] and have led to the updating of clinical practice guidelines [1, 13, 14]. The purpose of this study was to review the current evidence on the management of patients with an MPE in order to provide an updated view of the progress made in recent years.

## 2. Diagnostic Tests

### 2.1. Imaging Tests

A suspicion of PE can be radiographically confirmed at a volume of 200 mL. In addition, chest X-ray can detect as little as 50 mL of PF on a lateral view, which will show a costophrenic angle effusion [15]. A massive, loculated PE and a reduced volume of the ipsilateral lung are also suggestive of MPE. A retrospective study of more than 62,000 thoracocenteses for all-cause PE showed that chest ultrasound reduced the risk for pneumothorax by 19% (OR = 0.81; 95% CI = 0.74–0.90) [16]. Therefore, it is recommended that thoracic ultrasound, which has no associated complications, is performed prior to thoracentesis. Thoracic ultrasound can also suggest an MPE based on the finding of pleural thickening (>1 cm), diaphragmatic nodules or diaphragmatic thickening >7 mm, visceral pleura thickening, or pleural irregularity/nodularity [17].

Computerized tomography (CT) has high specificity but low sensitivity to separate benign PE from MPE, and mesothelioma from pleural metastasis [18]. In a recent study involving 370 patients, 211 (57%) had an MPE. CT identified 144 malignant PEs (sensitivity 68%; CI 95%: 62–75%). Of the 159 benign PEs, CT correctly classified 124 as benign (specificity 78%; CI 95%: 72–84%). The positive and negative predictive value of CT to identify MPE were 80% (CI 95%: 75–86%) and 65%, respectively (CI 95%: 58–72%). This means that one in three patients suspected to have an MPE will have it despite a negative CT scan [19].

To date, there is limited evidence on the usefulness of positron emission tomography (PET) to identify an MPE in routine clinical practice [20, 21]. This recommendation is based on the results of a recent meta-analysis involving 407 patients with an MPE and 232 patients with other types of effusions. This study yielded a moderate sensitivity and specificity for the diagnosis of malignancy (82% and 74%, respectively), as PET imaging will incorrectly miss disease in early stage tumours and incorrectly classify malignancy in conditions such as inflammatory pleuritis [20].

The role of PET-CT in MPE is still unclear. The results of the TARGET trial (ISRCTN14024829), aimed at assessing the diagnostic yield of PET-CT-guided pleural biopsy versus CT-guided pleural biopsy on suspicion of MPE, will shed some light on this issue [22].

Magnetic resonance (MR) offers better-quality images of soft tissue than CT scans. Therefore, MR has higher sensitivity to detect chest-wall and diaphragmatic involvement, although the lung images have a lower quality [23]. Although there are reports of sensitivity and specificity rates of 90% to differentiate malignant from benign PE, the optimal protocol has not yet been established. This added to limited access to MR in some centers has excluded MR from standard diagnostic tests for MPE.

### 2.2. Cytology of Pleural Fluid

Cytology is the initial test in establishing a diagnosis of MPE. The diagnostic performance of cytology is close to 60% [24], with very low sensitivity for mesothelioma (6%) and high for adenocarcinomas (79%) [25]. In a recent retrospective study involving 725 patients with a solid neoplasm and suspected pleural metastasis, PF cytology had a diagnostic performance of 63%. However, sensitivity varied according to the type of tumour and was lower for head and neck tumours and sarcomas (38% for the two types) and renal tumours (53%) and higher for breast (93%) and pancreatic cancer (100%) [26]. There are contradictory data on whether a high PF volume (>50–60 cc) can increase the diagnostic performance of cytology [27, 28]. However, it seems that a volume of 20–40 cc would be enough to optimize the yield [24]. The low diagnostic performance of this procedure is due to the fact that the tumour is not always located in the surface of mesothelial cells, where malignant cells will be exfoliated in PF, but they may also involve the layer below the serous layer; therefore, only a few malignant cells will reach the pleural space.

Immunohistochemistry can help differentiate reactive mesothelial cells from those of malignant pleural mesothelioma and adenocarcinoma metastases, exposing them to different antibody panels, as each of them has specific antigens. To validate the diagnosis of mesothelioma versus that of adenocarcinoma, two positive mesothelioma markers (anticalretinin, anticytokeratin CK5/6, anti-Wilms tumour antigen-1, or antiepithelial membrane antigen) and two negative markers for adenocarcinoma (anti-Ber-EP4, monoclonal anticarcinoembryonic antigen, antithyroid transcription factor-1, or anti-MOC-31) are required [29]. These markers can also establish the origin of adenocarcinoma with pleural metastases. Lymphocyte subtype analysis can identify hematological MPE in a specific cohort of patients with an undiagnosed PE [30].

### 2.3. Pleural Biopsy

Pleural biopsy is the gold standard procedure for the diagnosis of MPE [13]. Percutaneous pleural biopsy has a lower diagnostic sensitivity than cytology, as malignant parietal pleura infiltrates have a patchy pattern (46%) [31]. Its sensitivity, however, can reach 87–94% under ultrasound or CT guidance [32, 33]. In MPE with a pleural thickening >1 cm on chest X-ray, the diagnostic sensitivity of CT-guided pleural biopsy is similar to that of thoracoscopy (96% versus 95%) [34]. In addition, in an ultrasound-guided biopsy, tru-cut needles have better sensitivity than Abrams (70 versus 44%) [35]. When pleural thickening is millimetric or has difficult access, pleural biopsy can be performed by thoracoscopy (medical, under local anesthesia, or surgical under general anesthesia). The two procedures are useful to simultaneously visualize the pleura, perform a biopsy of the involved areas, and perform a PF drainage; in addition, if the pleura is infiltrated and in the absence of a trapped lung, pleurodesis can be performed.

Medical thoracoscopy is a safe procedure with low rates of complications and mortality and with a high diagnostic performance. It is very useful in patients not candidates for surgery or at increased risk of complications if more invasive procedures are used, such as video-assisted thoracoscopic surgery that requires general anesthesia [36]. The performance of these procedures (medical or surgical thoracoscopy) is similar [24, 37] and mortality and major complication rates are low (between 0–0.34% and 1.2–1.8%, respectively [5, 38, 39]).

### 2.4. Pleural Manometry

The indication of pleural manometry is to identify a nonexpandable lung through the calculation of pleural elastance (ability of the lung to return to its natural position after the extraction of PF). Its relevance is that the trapped lung (the lung does not reexpand to its normal position and the pleural elastance is elevated) is related to the failure of pleurodesis, since there is not a sufficient apposition between the pleural leaves. However, in the only randomized study conducted so far, *Lentz* et al. demonstrated that routine use of manometry during thoracentesis does not reduce chest discomfort related to the procedure and does not prevent against reexpansion pulmonary edema or pneumothorax ex vacuo [40]. Nevertheless, this data must be interpreted carefully. These measurements may not reflect the pressures of the pleural cavity, as they were only taken during brief interruptions in the drains. Determining the real pressures at the end of the expiration, which change several times during the respiratory cycle, is complex and may not be accurate [41].

### 2.5. Analysis of Pleural Fluid

PE can be a lymphocyte-rich exudate, although they can also be transudative [42]. PF is not associated with relevant biochemical characteristics, and determination of tumour markers has not conclusively proven to be useful. Analysis of PF was reported to have a general sensitivity of 54% in a study based on the determination of four markers (CEA, CA125, CA15-3, and cytokeratin [19]). In this study, cut-off points were established above the highest values observed in benign PEs. The authors concluded that these markers can be useful to identify the patients who will benefit the most from future invasive procedures for suspicion of MPE [43]. In general, the sensitivity and specificity of these markers are low [44], and they do not spare cytohistological confirmation.

Mesothelin is expressed in normal mesothelial cells and overexpressed in mesothelioma, lung, ovarian, and pancreatic cancer. Although mesothelin has been proposed as a marker for the diagnosis of mesothelioma [45], biomarker testing is recommended only for patients with a suspicious cytology who are not fit to undergo more invasive diagnostic testing [14].

Distinguishing reactive lymphocytes from hematopoietic malignant tumours may be challenging. In this setting, the diagnostic performance of PF flow cytometry is limited, and it should only be used in the presence of atypical cytological findings, a high clinical suspicion, or known history [46]. In PE secondary to multiple myeloma, a cytology or a monoclonal peak in PF have a very high diagnostic performance. Fluid is generally a lymphocytic-rich serohematic exudate with very high protein levels [47].

## 3. Management

The purpose of MPE therapies is to alleviate its symptoms, mainly dyspnea. Whereas asymptomatic effusions only require observation [2], the therapeutic options for symptomatic MPE include repeat therapeutic thoracentesis, thoracic drainage with pleurodesis, the insertion of an IPC, or surgery. The therapeutic choice should be made on a case-by-case basis, considering clinical factors and patient's preferences. Predicting the survival expectancy of a patient with MPE can contribute to a better therapeutic choice. Some tools have been developed and validated to assess the risk of mortality, such as the LENT score, based on lactate dehydrogenase in PF, the Eastern Cooperative Oncology Group performance score, the neutrophil/lymphocyte ratio, and type of tumour (Table 1), which estimates the risk of mortality based on the score obtained [48].

### 3.1. Therapeutic Thoracentesis

Therapeutic thoracentesis is recommended for all patients with an MPE involving 50% of the hemithorax and dyspnea. Although MPE can be caused by a variety of clinical problems, its symptoms generally improve after drainage. Subsequently, a definitive palliative intervention can be performed (IPC, pleurodesis, or both) [49]. In the absence of lung reexpansion, the treatment of choice is IPC to spare the patient the discomfort of recurrent unsuccessful pleurodesis. In patients with a life expectancy limited to a few days or weeks, repeated thoracentesis, up to a maximum of 1.5 L, may be performed to try to relieve symptoms (usually dyspnea, cough, or chest pain) [1, 3].

### 3.2. Thoracic Drainage and Pleurodesis

The purpose of pleurodesis is to induce an inflammatory response in the pleura that forces the adhesion of the two layers of the pleura to prevent the accumulation of fluid. The literature demonstrates that pleurodesis improves dyspnea, increases survival [50], and reduces the length of hospital stay and the need for future interventions [11, 51–59]. Pleurodesis is not recommended in the presence of a trapped lung (30% of cases) [60, 61], or multiple pleural septa, since the apposition of the two layers of the pleura will not be achieved and pleurodesis will be unsuccessful. The probability of success will increase if pH is <7.20 or effusion is > 50% of the hemithorax [62].

There are some aspects to be clarified, as the sclerosing agent to be used, the size of the drain tubes to be inserted, or the administration of nonsteroid anti-inflammatory agents to control pain.

Although the optimal agent for chemical pleurodesis has not yet been identified, talc is the most widely used agent for its availability and cost-effectiveness [49]. Talc can be administered in two forms: through the thoracoscope tube using an aerosol canister (talc poudrage) or via an intercostal tube as a suspension (talc slurry). Antibiotics (tetracycline, doxycycline, and bleomycin), bacterial agents (*Corynebacterium parvum*, OK432), or silver nitrate and iodopovidone have also been used. A recent meta-analysis of 62 randomized trials involving a total of 3,428 patients suggests that talc insufflation is the most effective method of pleurodesis in preventing the accumulation of fluid. However, the clinical and statistical heterogeneity and the high risk of bias in most of the studies included make further research necessary, to confirm that talc poudrage is more effective that talc slurry and doxycycline [4]. Regardless of whether the sclerosing agent is insufflated via a chest tube or the thoracoscope, hospitalization is required. Whereas insufflation through a chest tube can be performed at bedside with analgesia, the second procedure requires general anesthesia or conscious sedation. Randomized clinical trials have failed to demonstrate the superiority of a technique over the other [60, 63], and the British Thoracic Society reports that the efficacy of the two procedures is similar [3]. In a recent study, Bhatnagar et al. compared the efficacy of talc administration of talc poudrage by thoracoscopy under local anesthesia versus talc slurry administered through a chest drain. The rate of pleurodesis failure at 90 days in patients who received talc poudrage was 22% (36/161) versus 24% (38/159) for the patients who were given talc slurry (OR 0.91; 95% CI: 0.54–1.55; *p*=0.74). Although no significant differences were observed between the two groups, the authors acknowledged that their study had limited power to detect small—albeit potentially relevant—differences [64].

Although a study yielded a rate of success of pleurodesis of 91% [65], these results have not been attained in other studies. A randomized trial comparing talc poudrage versus talc slurry reported success rates of 71% and 78%, respectively. However, when patients who died within 30 days or failed to achieve lung reexpansion were included, success rates decreased to 53% and 60%, respectively [60]. In a recent study, the overall rate of success of pleurodesis was 81.4% (84.9% for nonmesotheliomas and 73% for mesotheliomas). The latter may be explained by the lack of normal pleural tissue susceptible of an inflammatory response [66]. When talc is instilled, it is recommended to instill large-particle talc (>15 *μ*m) to prevent the development of acute respiratory distress syndrome [67, 68]. Fever and chest pain are other complications of intrapleural talc [12].

The size of the drainage tube is another matter of controversy. Previous studies had documented a similar rate of success for small-bore chest tubes (10–14 F) and large-bore chest tubes when used to insufflate a sclerosing agent [69–71]. However, the TIME-1 trial suggests that large-bore tubes are superior to small-bore tubes in performing pleurodesis [11]. Moreover, small-bore chest tubes are more comfortable for patients [12]. The same study revealed that the administration of nonsteroidal anti-inflammatories to control pain does not affect pleurodesis outcomes [12]. No clinical data have been published on the potential deleterious effects of the administration of corticosteroids on the efficacy of pleurodesis. Therefore, their use should be avoided whenever it is possible. In addition, rotation of the patient has not been demonstrated to improve the rate of successful pleurodesis [72].

### 3.3. Indwelling Pleural Catheter

**Figure 1:**
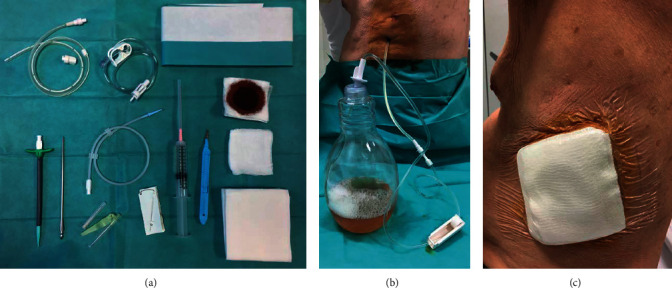
(a) Example of indwelling pleural catheter set. (b) Drainage of indwelling pleural catheter with vacuum bottle. (c) IPC with dressing.

IPC are silicone tubes that are inserted percutaneously. They have a one-way valve and maintain lung expansion by intermittently draining pleural fluid instead of inducing pleural space obliteration, as in the case of pleurodesis. The purpose of IPC is to control symptoms without hospitalization (Figure 1). IPCs are as effective as pleurodesis as a first-line treatment of MPE [11, 59] and can be used in the presence of a trapped lung. A systematic review of 19 studies involving 1,370 patients assessing the efficacy and safety of IPC for MPE revealed an improvement of symptoms in 95% of cases [73]. IPC can achieve spontaneous pleurodesis in 46–70% of patients with full lung expansion caused by local inflammation induced by the tumour or the IPC [73, 74].

**Figure 2:**
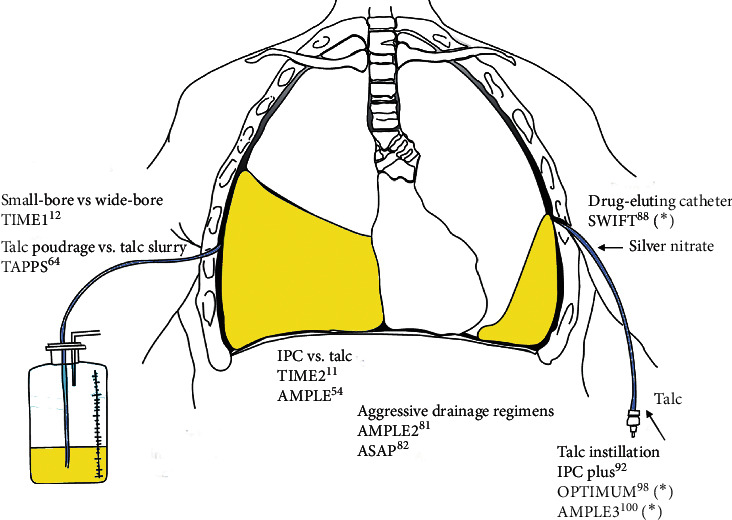
Recent trials addressing the management of malignant pleural effusion (modified under authorization) [41]. IPC, indwelling pleural catheter. ^*∗*^In recruitment phase.

To date, no supporting evidence has been provided on the superiority of a technique over another (IPC versus talc pleurodesis). IPC requires a shorter hospital stay and less repetition of pleural procedures [75] and is more effective in the presence of a trapped lung or in patients with a poor functional status that cannot tolerate pleurodesis. In five studies involving 133 patients with MPE and a trapped lung, symptoms improved by more than 94% in patients with an IPC [76–80]. The AMPLE study compared the length of hospital stay from the procedure (IPC versus talc pleurodesis) until death or at 12 months in 146 patients with MPE. In the first group, the median hospital stay was 10 days (interquartile range 3–17) versus 12 (7–21) in the second group (*p*=0.03). Although the clinical relevance of this difference is uncertain, these findings may guide the therapeutic decision [54]. PE can be more rapidly solved by pleurodesis, but it is more invasive and will probably require recurrent aspiration [11, 59]. IPC is more suitable for ambulatory patients, although drainage by IPC is more time-consuming and requires more intensive care in patients who have undergone unsuccessful pleurodesis. The two procedures are effective in achieving the relief of symptoms and improving quality of life, without significant differences between the two. The AMPLE-2 trial, which involved 87 patients with an MPE treated with an IPC, compared the optimal drainage protocol (daily or only in the presence of symptoms). No differences were found between the two techniques in terms of dyspnea control, but a higher rate of success of pleurodesis at 2 and 6 months was achieved with the more invasive drain method [81]. The results of this study are consistent with the ones of the ASAP trial [82].  Figure 2 contains a summary of the trials on this issue that have been published or are in recruitment phase.

IPC are associated with higher complication rates (drain blockade or malposition (<5%) [83], catheter rupture [84], and subcutaneous and pleural infections (0–12%)) [85–87], although they are generally well tolerated without a significant morbidity [59]. Another potential complication related to the use of IPC is tumour seeding of the catheter tract, which occurs in 5% of cases based on the type of tumour and time of use of the IPC [86]. Prophylactic use of radiotherapy has not been documented to exert any beneficial effect and routine use in clinical practice is not recommended [88].

Little evidence has been published of the cost of the management of IPC/talc pleurodesis, as most studies are retrospective and perform an indirect comparison with conventional treatments. The TIME-2 study, carried out prospectively, did not reveal any differences between these techniques in terms of cost [89]. A cost-effectiveness analysis performed alongside the TIME2 trial suggested that IPC is cost-effective compared to talc: to a greater extent in patients with limited survival and less so if significant nursing resources are required to assist with weekly drainage [90].

IPC can be more cost-effective in patients with a limited life expectancy (<3 months), whereas talc pleurodesis is more cost-effective in patients with a higher life expectancy [91].

### 3.4. Associated Procedures

The combination of several treatments, especially IPC chemical pleurodesis, is a plausible alternative in an attempt to reduce the length of hospitalization and time of catheter use. In a recent study, 154 patients with MPE were implanted an IPC, and PF was evacuated in ambulatory care. Patients with a trapped lung were randomized to receive either talc or placebo via the IPC. The results showed that pleurodesis was achieved in 43% (30/69) of patients who were administered talc, and in 23% (16/70) of subjects in the placebo group (HR 2.20; 95% CI: 1.23–3.92; *p*=0.008), without any adverse effects [92].

### 3.5. Other Intrapleural Treatments

Two randomized phase II studies investigated the use of a monoclonal antibody, bevacizumab, intrapleurally. In the first, performed in patients with non-small cell lung cancer and MPE, the treatment arm with this drug obtained a greater reduction in the size of PE and symptoms, with a higher survival rate per year of follow-up. Adverse events were similar among the groups [93]. In the second, performed in patients with nonsquamous non-small cell lung cancer and MPE, better MPE response rates were observed with the addition of bevacizumab [94]. Also, in malignant pleural mesotheliomas, the role of gene therapy and immunotherapy can change the management of these patients [95]. However, more clinical trials are needed to determine the efficacy of these treatments.

In a recent clinical trial, 71 patients with an MPE who did not drain were randomized to receive urokinase or placebo. No differences were found between the groups in terms of dyspnea and the failure of pleurodesis (37% and 32%, respectively). In contrast, the group receiving urokinase observed a greater decrease in PE size (−19% versus −11%; *p* < 0.001), a lower hospital stay (1.6 daysversus 2.6; p.049) and improved survival (69 days versus 48; *p*=0.026). Their conclusions are that use of intrapleural urokinase does not reduce dyspnea or improve pleurodesis success compared with placebo and cannot be recommended as an adjunct to pleurodesis [96].

### 3.6. Surgery

Surgical pleurectomy has a limited role in the management of MPE, and the supposed benefits may not outweigh the perioperative mortality and worsening of quality of life [3]. A study on mesothelioma-related MPE revealed that talc pleurodesis may be preferable to surgery, as it is associated with lower rates of complications and a shorter length of hospitalization [97].

### 3.7. Future Clinical Trials

Three trials on this issue are ongoing at this moment, the OPTIMUM trial (comparing the improvement in quality of life achieved by IPC talc pleurodesis versus chest-tube talc pleurodesis) [98]; the SWIFT trial (comparing the rates of successful pleurodesis via silver nitrate-coated IPC versus standard IPC) [99]; and the AMPLE3 trial [100] (comparing the advantages of IPC and talc pleurodesis versus video-assisted thoracoscopy); and MESOTRAP [101] (randomised controlled clinical trial comparing video-assisted thoracoscopic partial pleurectomy or decortication with IPC in patients with trapped lung and malignant pleural mesothelioma).  Table 2 shows a summary of the main trials that evaluate the management of MPE with the results obtained.

## 4. Conclusions

The optimal management of patients with MPE is still unclear. This problem requires a multidisciplinary approach. Less-invasive diagnostic tests are the initial step. If results are negative, more invasive techniques are required taking into account patient's preferences, their functional status and life expectancy, the type of tumour, experience of the medical team, or the presence of a trapped lung. At present, the management of pleural effusion has evolved towards less invasive approaches based on ambulatory care. This approach spares the patient the discomfort caused by more invasive interventions and reduces its economic burden.

**Figure 3:**
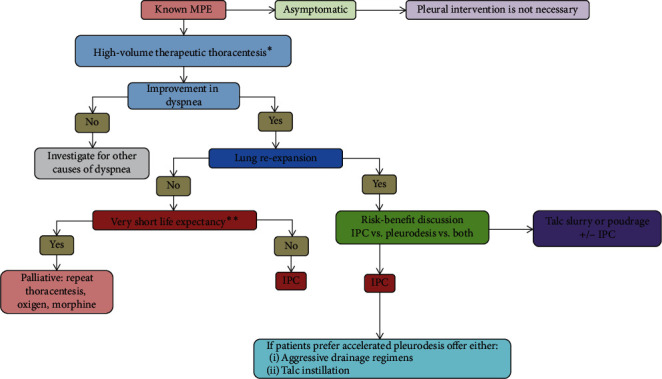
Management of patients with known malignant pleural effusion (adapted from Feller-Kopman et al.) [1]. IPC, indwelling pleural catheter; MPE, malignant pleural effusion. ^*∗*^With the aim of evaluating lung expansion and improvement of dyspnea. ^*∗∗*^In an approximate and individualized fashion.

The evidence currently available on the management of symptomatic MPE suggests that both, talc pleurodesis (poudrage or slurry) and IPC, are effective in reducing fluid accumulation and in the relief of symptoms (Figure 3). Limited data has been provided demonstrating the superiority of one over another (poudrage versus slurry). Although IPC reduces the length of hospitalization, long-term use is associated with a higher risk for adverse events. In the presence of a trapped lung, IPC is more effective in improving symptoms. In the case of loculated MPE, the use of intrapleural fibrinolytics is not supported by evidence, and oncologic treatments are not currently recommended as an alternative option to mechanical drainage. Further research is needed to shed light on the current matters of controversy such as performing pleurodesis with less invasive techniques or being able to offer each patient a personalized approach to managing their MPE, based on variables (e.g., symptoms, type of cancer, or size of PE) that could predict the individual response and prognosis of each patient.

## Figures and Tables

**Table 1:** A prognostic score for malignant pleural effusions (LENT) [33].

	Variable	Score
L	*LDH in pleural fluid*	
	<1,500 UI/L	0
	>1,500 UI/L	1

E	*ECOG performance score*	
	0	0
	1	1
	2	2
	3-4	3

N	*Neutrophil/lymphocyte ratio*	
	<9	0
	>9	1

T	*Type of tumour*	
	Low risk (mesothelioma and hematologic malignancies)	0
	Moderate risk (breast cancer, gynecological, and renal cell carcinoma)	1
	High risk (lung cancer and other tumours)	2
	*Risk by category*	*Total score*
	Low risk (median of survival: 319 days)	0-1
	Moderate risk (median of survival: 130 days)	2–4
	High risk (median of survival: 44 days)	5–7

ECOG, eastern cooperative oncology group; LDH, lactate dehydrogenase.

**Table 2:** Main trials investigating management of malignant pleural effusion.

Authors and references	Year of publication	Comparator	Commentary
Davies et al. [11] (TIME2)	2012	IPC versus talc pleurodesis	No significant differences between the two groups to relieve dyspnea
Rahman et al. [12] (TIME1)	2015	Use of nonsteroidal anti-inflammatory drugs (NSAID) and chest drain size	NSAID versus opiates was associated with more need for rescue medication in the first with no lower rates of pleurodesis efficacy. The 12 F versus 24 F chest tubes were associated with pain reduction but did not meet the noninferiority criteria for the efficacy of pleurodesis.
Thomas et al. [54] (AMPLE)	2017	IPC versus talc pleurodesis	Median hospitalization days was lower in IPC patients (*p*=0.03)
Wahidi et al. [82] (ASAP)	2017	IPC: Daily drainage versus symptom-guided drainage	Higher success rate of pleurodesis with aggressive drainage, without improving control of dyspnea
Muruganandan et al. [81] (AMPLE2)	2018	IPC: Daily drainage versus symptom-guided drainage	Higher success rate of pleurodesis with aggressive drainage, without improving control of dyspnea
Bhatnagar et al. [92] (IPC plus)	2018	Talc pleurodesis through IPC versus placebo	Pleurodesis in 43% and 23%, respectively (*p*=0.008)
Mishra et al. [96] (TIME3)	2018	Intrapleural urokinase versus placebo	Urokinase does not reduce dyspnea or improve pleurodesis success rate
Bhatnagar et al. [64] (TAPPS)	2019	Talc *poudrage* by thoracoscopy versus talc *slurry* by chest drainage	Pleurodesis failure rate of 22% in the first group and 24% in the second (*p*=0.74)
Sivakumar et al. [98] (OPTIMUM)	—^*∗*^	Talc pleurodesis through IPC versus chest drainage (small size)	Improving the quality of life will be assessed
ClinicalTrials.gov identifier: NCT02649894 [99] (SWIFT)	—^*∗*^	Silver nitrate eluting catheter versus standard IPC	The success rate of pleurodesis will be assessed
Anzctr.org.au identifier: ACTRN12618001013257 [100] (AMPLE3)	—^*∗*^	IPC versus VATS pleurodesis	Requirement for ipsilateral pleural procedure
Matthews et al. [101] (MESOTRAP)	—^*∗*^	VATS-PD versus standard IPC	Improvement of dyspnea will be assess in patients with trapped lung and malignant pleural mesothelioma

^*∗*^Unpublished results; VATS, video-assisted thoracoscopy surgery; VATS-PD, video-assisted thoracoscopic partial pleurectomy or decortication.

## Abbreviations

CT: Computed tomography
IPC: Indwelling pleural catheter
MPE: Malignant pleural effusion
MR: Magnetic resonance
PE: Pleural effusion
PET: Positron emission tomography
PF: Pleural fluid.
